# The Effect of PM_10_ on Allergy Symptoms in Allergic Rhinitis Patients During Spring Season

**DOI:** 10.3390/ijerph120100735

**Published:** 2015-01-13

**Authors:** Il Gyu Kang, Youn Hee Ju, Joo Hyun Jung, Kwang Pil Ko, Dae Kyu Oh, Jeong Hee Kim, Dae Hyun Lim, Young Hyo Kim, Tae Young Jang, Seon Tae Kim

**Affiliations:** 1 Department of Otolaryngology, Gil Medical Center, School of Medicine, Gachon University, Incheon 405-760, Korea; E-Mails: eyik@naver.com (I.G.K.); erica3743@naver.com (Y.H.J.); d0ramong@hanmail.net (J.H.J.); 2 Department of Preventive Medicine, School of Medicine, Gachon University, Incheon 406-799, Korea; E-Mails: kpko@gachon.ac.kr (K.P.K.); jsodk@hanmail.net (D.K.O.); 3 Environmental Health Center for Allergic Rhinitis, Inha University Hospital, Ministry of Environment, Incheon 400-711, Korea; E-Mails: kimjhmd@inha.ac.kr (J.H.K.); dhyunlim@inha.ac.kr (D.H.L.); 4 Department of Pediatrics, School of Medicine, Inha University, Incheon 400-711, Korea; 5 Department of Otorhinolaryngology-Head and Neck Surgery, Inha University College of Medicine Incheon 400-711, Korea; E-Mails: inhaorl@naver.com (Y.H.K.); jangty@inha.ac.kr (T.Y.J.)

**Keywords:** air pollution, particulate matter, allergic rhinitis

## Abstract

*Background*: Asian sand dust (ASD) that originates in the Mongolian Desert in the spring induces serious respiratory health problems throughout East Asia (China, Korea, Japan). PM_10_ (particulate matter with an aerodynamic diameter <10 μm) is a major air pollutant component in ASD. We studied the effects of PM_10_ on allergy symptoms in patients with allergic rhinitis during the spring season, when ASD frequently develops. *Methods*: We investigated the changes in allergic symptoms in 108 allergic patients and 47 healthy subjects by comparing their 120-day symptom scores from February to May 2012. At the same time, the contributions of pollen count and PM_10_ concentration were also assessed. We also compared symptom scores before and 2 days after the daily PM_10_ concentration was >100 μg/m^3^. Results: The PM_10_ concentration during the 120 days was <150 μg/m^3^. No significant correlations were observed between changes in the PM_10_ concentration and allergic symptom scores (*p* > 0.05). However, allergic symptoms were significantly correlated with outdoor activity time (*p* < 0.001). *Conclusions*: These results demonstrate that a PM_10_ concentration <150 μg/m^3^ did not influence allergy symptoms in patients with allergic rhinitis during the 2012 ASD season.

## 1. Introduction

Asian sand dust (ASD) particles are an important air pollutant material that originates in East Asia from China and Mongolian Desert storms during the spring season (February–May) [1,2]. Additionally, most ASD particles include minerals and microorganisms [2]; however, they also include many pollutants, particularly particulate matter <10 μm in aerodynamic diameter (PM_10_). Moreover, the PM_10_ concentration accounts for 53%–70% of total ASD particulate matter [3,4]. PM_10_ particles are the major cause of respiratory system inflammatory reactions [5,6,7].

The association between dust events and death from cardiovascular and respiratory causes is statistically significant for all pollutants [8,9,10]. It has also been suggested that patients with advanced respiratory disease might be more susceptible to ASD events [11,12]. ASD stimulated chemical mediators and mucin production in an allergic murine model [13,14,15]. This allergic inflammation was activated by mineral elements (mainly SiO_2_), which increases interleukin-5 and monocyte chemotactic protein-3 expression levels [16]. ASD also enhanced allergic reactions in guinea pigs repeatedly administered Japanese cedar pollen particles [17]. PM_2.5_ (fine particles with aerodynamic diameter < 2.5 μm) may enhance allergic sensitization through interactions with allergens [18]. Many reports have demonstrated a relationship between air pollution and exacerbation of asthma and other allergic diseases [19,20,21]. About 30% of adult patients with asthma show worsening of upper and/or lower respiratory, ocular, or cutaneous symptoms during ASD events [22,23].

Although some reports have suggested a possible negative effect of ASD on allergic diseases [24,25], no reports have determined whether ASD PM_10_ influences allergy symptoms in patients with allergic rhinitis. In this study, we clarified the effects of PM_10_ and pollen concentrations on allergy symptoms of patients with allergic rhinitis during the spring ASD season.

## 2. Patients

We evaluated 108 patients with allergic rhinitis and 47 controls without allergic rhinitis at the Gachon University Gil Medical Center and the Inha University Hospital. A total of 108 allergic patients, who were previously diagnosed and treated for allergic rhinitis with positive skin tests and Immunocap^®^ tests for *Dermatophagoides pteronysinus* and *Dermatophagoides farine*, were enrolled. The allergic rhinitis severity level in these patients was classified into four groups according to criteria of the 2009 Allergic Rhinitis Impact on Asthma (ARIA) guidelines (I: mild intermittent, II: moderate to severe intermittent, III: mild persistent, and IV: moderate to severe persistent). We enrolled 47 volunteers as a control group and confirmed that they had no allergies by clinical history assessments, skin tests, and physical examinations. This study was approved by the institutional review boards from both institutions.

## 3. Methods

The allergy patient and control groups recorded their symptoms in a daily symptom diary. They checked for allergy symptoms by assessing rhinorrhea, nasal obstruction, sneezing, itching, and sleep disturbance levels using a modified six-point Likert scale (0: no symptoms, 5: most serious symptoms) for 120 days from 1 February to 30 May 2012 [26]. We then evaluated the serial correlations between the symptom scores and PM_10_ changes over 120 days (long-term observations). We also evaluated symptom changes during the 2 days before and after the 3 event days, when the daily PM_10_ concentrations peaked at >100 μg/m^3^ (short-term observations). The subjects also recorded their outdoor activity time in their diaries. The guidelines established by the National Health Environmental Research Center suggest that the sensitive group (the airway and cardiac disease patients) could be influenced by PM_10_ concentrations of 81–120 μg/m^3^. A questionnaire that assessed life quality, comorbid diseases, and ARIA levels was also evaluated.

**Figure 1:**
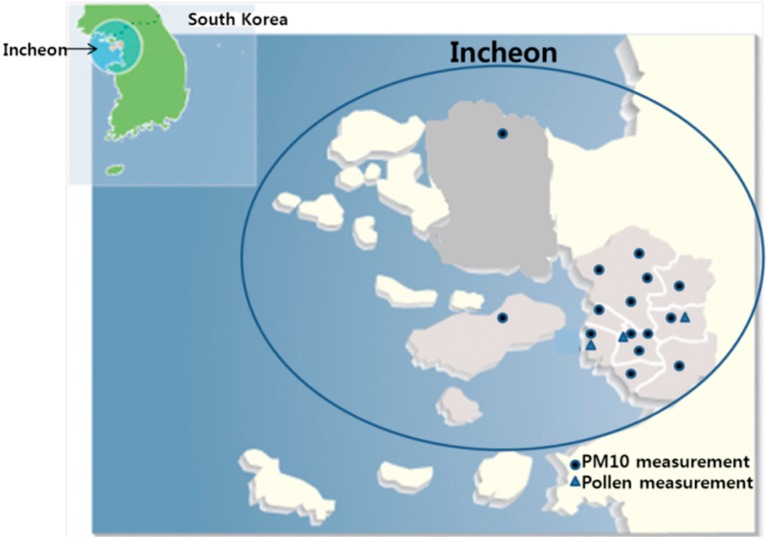
Map of the monitoring station locations and basic area features. Circle: PM_10_ measurement area, Triangle: pollen measurement area.

PM_10_ concentrations were evaluated in 10 areas of Incheon City using information made public by the Incheon City Health Environmental Research Center (Table 1). This center publishes monthly data for five major air pollutants (PM_10_, PM_2.5_, SO_2_, O_3_, CO, and NO_2_). Pollen concentrations were also evaluated in three areas inside Incheon City, including a number of tree and herb pollens (Needle Fir, Japanese Maple, Japanese Chestnut, Wind Spindle Tree, Chinese Bayberry, Japanese Red Pine, Oak, Korean Willow, Ragweed, Wormwood, Rice, and Trumpet Lily, Figure 1).

**Table 1:** **P**M_10_ concentrations measured in 15 areas of Incheon City.

Mar Day	1	2	3	4	5	6	7	8	9	10	11	12	13	14	15
25	64	63	62	79	52	55	59	66	76	55	58	60	78	73	68
26	46	49	50	52	38	39	43	43	61	50	42	32	41	47	42
27	56	56	55	56	49	46	47	48	69	59	49	43	45	54	50
28	157	159	151	144	129	143	116	115	188	146	141	109	126	138	135
29	159	146	127	113	126	125	105	89	171	152	129	87	99	106	109
30	56	63	60	49	45	55	53	56	73	60	55	40	40	49	39
31	78	74	81	107	70	69	80	89	92	77	71	59	98	56	47

Notes: PM_10_ concentrations, μg/m^3^, PM_10_, particulate matter <10 μg in aerodynamic diameter.

These data were supplied by the Environmental Health Center for Allergic Rhinitis (Inha University Hospital). They used a 7-day recording volumetric spore sampler (Burkard Manufacturing Co., Ltd., Hertfordshire, UK). Pollen was counted as the number of pollen particles in 1 m^3^ using Pan-American Aerobiology Association standardized protocols.

### Statistical Analyses

The patient’s characteristics are described as proportions. We used a mixed regression model to evaluate the association between PM_10_ concentrations and allergy symptoms, which were measured consecutively for 120 days in allergic patients and normal subjects (analysis for long-term observations). We also used a mixed regression model to evaluate which factors, including pollen counts and time spent outdoors, were associated with allergy symptoms in allergic patients. In the mixed regression model, we corrected for within-subject covariance using a first-order autoregressive covariance structure. When there were days with >100 μg/m^3^ PM_10_ concentration (event days), we compared allergy symptom scores recorded before those 2 days with scores recorded after the event day in allergic patients and normal subjects using repeated-measures analysis of variance for a 120-day observation period (analysis for short-term observations). We conducted a correlation analysis with mean allergic nasal symptoms and PM_10_ concentrations with lag times (0, 1, 2 days) from event day to assess the relationship between the most-affected days after the event day and allergic nasal symptoms. All analyses were conducted using SAS 9.3 (SAS Institute, Cary, NC, USA).

## 4. Results

### 4.1. Patient Characteristics

We selected 108 allergic patients (58 male, 50 female) and 47 controls (19 male, 28 female) The average patient age was 20 years. No differences in age and sex distributions were observed between the patient and control groups. The main symptoms for the allergic patients were rhinorrhea, sneezing, nasal obstruction, and sleep disturbance. According to the ARIA guidelines, the mildly persistent patients were the most common group (Table 2). ARIA class I: mild intermittent symptoms, II: moderate to severe intermittent symptoms, III: mildly persistent symptoms, IV: moderate to severe persistent symptoms.

### 4.2. PM_10_ and Pollen Count Measurements

**Figure 2:**
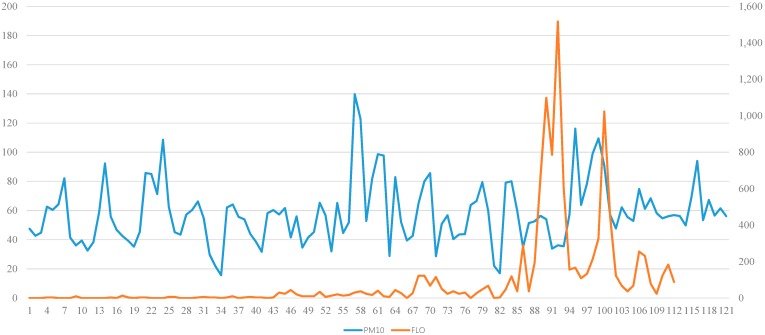
PM_10_ and pollen dispersions. The PM_10_ and pollen concentrations were measured consecutively for 120 days. PM_10_: PM_10_ concentration; FLO: pollen concentration. (Lt. bar: PM_10_ μg/m^3^; Rt bar unit: pollen particle/m^3^).

**Figure 3:**
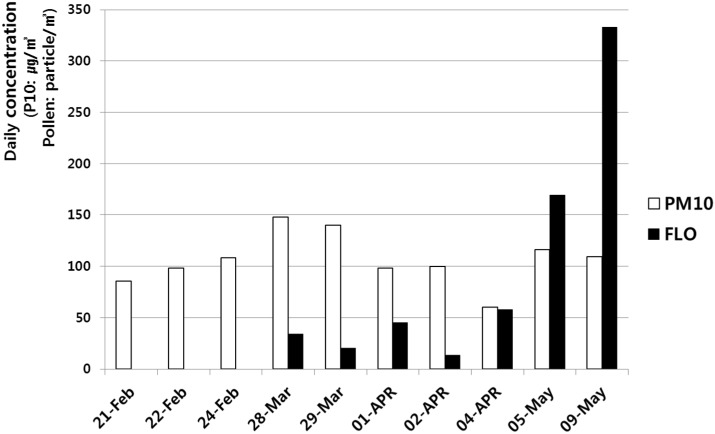
PM_10_ and pollen dispersions. The three event days when the PM_10_ was >100 µg/m^3^ were 24 February, 29 March, and 5 May. PM_10_, PM_10_ concentration; FLO, pollen concentration.

We measured PM_10_ concentrations continuously for 120 days (Figure 2). In the past, the PM_10_ concentration increased to >400 μg/m^3^ for an average of 10 days during the ASD season (February–May); however, in 2012, PM_10_ concentration did not rise that high. Specifically, the highest PM_10_ concentration in 2012 was <150 μg/m^3^. We evaluated the three event days when the PM_10_ concentration was >100 μg/m^3^. These three event days were 24 February (105.53 μg/m^3^), 29 March (139.8 μg/m^3)^, and 5 May (116.13 μg/m^3^). Additionally, pollen counts increased very significantly in May compared with those in February and March (Figure 3).

**Table 2:** Patient characteristics.

Characteristics	Allergic Rhinitis Patients
Male/Female	58/50
Age	6–12: 28.7%
	13–18: 17.7%
	20–29: 22.5%
	30–39: 14%
	≥40: 17.1%
ARIA class	I: 9.4% , II: 14.1%, III: 69.5%, IV: 7%
Associated disease	Atopic dermatitis: 28.8%
	Asthma: 11.1%
	Sinusitis: 29.6%
Allergy-related familial history	54.4%

### 4.3. The Effect of PM_10_ on Allergy Symptoms

We collected and classified all types of pollen (Table 3). The long-term observations demonstrated that the daily PM_10_ changes were not significantly correlated with changes in allergy symptoms, including nasal obstruction (*p* = 0.6137), rhinorrhea (*p* = 0.9427), sneezing (*p* = 0.9032), itching (*p* = 0.1536), sleep disturbance (*p* = 0.5946), or total symptom score (*p* = 0.6176). No significant changes were observed in the control group. These results demonstrate that the nasal symptoms of the patients with allergy were not influenced by PM_10_ concentrations <150 μg/m^3^ during the spring season (Table 4). However, significant correlations between total nasal symptom scores and outdoor activity time (*p* < 0.001) was observed. These data indicate that allergy symptoms were significantly aggravated by an increase in outdoor exposure time. Temperature had a significant effect in both groups (Table 5).

**Table 3:** Pollen counts. Total pollen counts were measured in three areas of Incheon city.

Pollen	Scientific Name	Genus Name
Tree	Abies	Needle Fir
	Acer	Japanese Maple
	Castanea	Japanese Chestnut
	Euonymus	Wind Spindle Tree
	Myrica	Chinese Bayberry
	Pinus	Japanese Red Pine
	Quercus	Oak
	Salix	Korean Willow
	Ambrosia	Ragweed
Herb	Artemisia	Wormwood
	Gramineae	Rice
	Lilyaceae	Trumpet Lily

**Table 4:** The effects of PM_10_ on allergy symptoms according to the long-term observations (*p*-value).

Effect		Estimate	Standard Error	*p*-value
Rhinorrhea	allergy group	−0.00041	0.000306	0.1787
	control group	−0.000018	0.000254	0.9425
Itching	allergy group	−0.00037	0.000294	0.1201
	control group	0.00010	0.000163	0.5210
Nasal Obstruction	allergy group	−0.00008	0.000320	0.7948
	control group	−0.00023	0.000340	0.4951
Sneezing	allergy group	−0.00033	0.000311	0.2240
	control group	−0.00005	0.000276	0.8623
Sleep disturbance	allergy group	−0.00037	0.000213	0.0809
	control group	0.000047	0.000111	0.6729
Total symptom score	allergy group	−0.00160	0.000884	0.0694
	control group	−0.00069	0.000724	0.3377

**Table 5:** The relationships of pollen concentration and time outside with total nasal symptom scores.

Effect	Allergy Group			Control Group		
	Estimate	Standard error	*p*-value	Estimate	Standard error	*p*-value
FLO	−0.00028	0.000172	0.1015	−0.00004	0.000083	0.6335
OUT	0.1243	0.007797	<0.001	0.05990	0.007874	<0.0001
HUMID	−0.00055	0.001404	0.6973	−0.00114	0.001249	0.3620
TEMP	−0.00575	0.007319	0.4318	−0.01008	0.006282	0.1087

Notes: FLO, pollen concentration; Out, time outside; Humid, humidity; Temp, temperature.

No specific changes in the allergy symptom scores before and after the event days were detected in the short-term observations (event days) (Table 6).

**Table 6:** The effects of PM_10_ on allergy symptoms and drug use according to the short-term observations (*p*-value).

Effect	24 February	29 March	5 May
Rhinorrhea	0.88	0.41	0.72
Itching	0.88	0.67	0.24
Nasal obstruction	0.19	0.65	0.52
Sneezing	0.19	0.66	0.19
Sleep disturbance	0.67	0.72	0.48
Total nasal score	0.53	0.95	0.15
Drug use	0.49	0.53	0.49

We also investigated the number of most-affected days after high PM_10_ concentration exposures. Compared with lag0 (the increased day) symptoms, the lag1 (the next day) and lag2 (day 2) symptoms were not aggravated (Table 7).

**Table 7:** The relationship between the most-affected days after high PM_10_ concentration exposures and allergic nasal symptoms.

Days	Rhinorrhea	Sneezing	Nasal Obstruction	Itching	Sleep Disturbance	Total Score
**Lag0**						
*p*-value	0.744	0.704	0.747	0.578	0.603	0.888
Correlation	0.030	0.035	−0.030	−0.051	−0.048	−0.013
**Lag1**						
*p*-value	0.937	0.567	0.642	0.924	0.979	0.717
Correlation	−0.007	−0.053	−0.043	−0.009	−0.002	−0.033
**Lag2**						
*p*-value	0.902	0.482	0.432	0.837	0.793	0.658
Correlation	0.011	−0.065	−0.073	0.019	−0.024	−0.041

Notes: Lag0: increased PM_10_ day; Lag1: day after increased PM_10_; Lag2: two days after increased PM_10_.

No differences in the change in symptoms were observed according to ARIA classification (data not shown). Additionally, children (subjects <13 years old) who were more sensitive did not have different total symptom scores compared with older subjects. Moreover, no differences were detected between the sexes (Table 8).

**Table 8:** The relationship between total symptom scores, age (<13 years) and sex.

Effect	Estimate	Standard Error	*p*-value
PM_10_	−0.00217	0.001398	0.1198
Age	0.01169	0.2142	0.9567
Sex	0.05494	0.8565	0.9492

## 5. Discussion

The PM_10_ concentration frequently increases during the spring ASD season in Korea, and we hypothesized that increased PM_10_ during the ASD might play a role in aggravating allergy symptoms. PM_10_ concentrations have increased >400 μg/m^3^ in the past 10 years, and most people have experienced aggravated respiratory symptoms during the ASD season. However, our results demonstrate that PM_10_ concentrations <150 μg/m^3^ did not aggravate allergy symptoms in patients with allergic rhinitis.

A significant increase in the variation in pulmonary function has been observed during Asian dust days compared with control days [11,24]. Additionally, exercise-induced bronchial reactivity, atopic asthma, and skin prick tests positive for indoor allergens increased significantly along with PM_2.5_ concentrations in primary school children [18]. Personal PM_2.5_ levels in asthmatic allergic children living in urban areas are correlated with the percentages of nasal eosinophils [19]. Additionally, children living <50 m from a heavily trafficked road were more likely to develop asthma. This study also reported a possible link between developing asthma and increased PM_10_ concentration. However, the International Study of Asthma and Allergies in Childhood (ISAAC) reported a weak negative association between PM_10_ and various outcomes [25]. These findings suggest that the urban PM_10_ background has little or no association with the prevalence of childhood asthma, rhinoconjunctivitis, or eczema [26]. In contrast to previous studies, our data show similar results to those of the ISAAC study.

In another study, subjects reported significantly higher respiratory symptom frequency during Asian dust days compared with that during control days. The effects of dust storms on asthma admissions were prominent two days after the event (8%); however, this association was not statistically significant [27]. In our study, we evaluated the effects of PM_10_ on allergy symptoms two days before and again after event days, and we found that increased PM_10_ did not influence the changes in allergy symptoms. This result suggests that allergic patients were not affected by PM_10_ concentrations <150 μg/m^3^. ASD concentrations frequently increase to 400–800 μg/m^3^ during the ASD season in Korea; however, the PM_10_ concentration during the current season was not sufficient to stimulate allergy symptoms.

Our data demonstrate that outdoor activity time was significantly correlated with allergic symptom scores. This result suggests that outdoor exposure time has a more meaningful impact on allergy symptoms than does PM_10_ concentration. These results also emphasize that we cannot suggest that pollen and PM_10_ concentrations have a synergistic effect aggravating symptom scores.

We hypothesized that those with higher ARIA grade might be more influenced by the increased PM_10_ concentration. However, our data did not show a positive correlation with the ARIA grades. The PM_10_ concentration in this study was associated with a suboptimal level that is capable of inducing symptom changes in most symptomatic patients with allergic rhinitis.

## 6. Conclusions

Our data demonstrated no correlation between PM_10_ and allergy symptom scores in patients with allergic rhinitis when the PM_10_ concentration was <150 μg/m^3^.
